# Phenolic enrichment of foods curated in olive oil: Kinetics and chemical evaluation

**DOI:** 10.1016/j.fochx.2024.101398

**Published:** 2024-04-22

**Authors:** A. Castillo-Luna, F. Priego-Capote

**Affiliations:** a Department of Analytical Chemistry, Campus of Rabanales, University of Córdoba, Córdoba, Spain; b Chemical Institute for Energy and Environment (IQUEMA), Campus of Rabanales, University of Córdoba, Córdoba, Spain; c Maimónides Institute of Biomedical Research (IMIBIC), Reina Sofía University Hospital, University of Córdoba, Córdoba, Spain; d Agrifood Campus of International Excellence (ceiA3), University of Cordoba, Córdoba, Spain; e Consortium for Biomedical Research in Frailty & Healthy Ageing, CIBERFES, Carlos III Institute of Health, Spain

**Keywords:** Foods, Virgin olive oil, Curation, Phenolic compounds, Enrichment, Secoiridoids

## Abstract

Since ancient times food has been preserved in vegetable oils for curation. Nevertheless, the transfer of bioactive compounds from these oils to curated foods has not been studied. This research has evaluated the phenolic enrichment of foods curated in olive oil. For this purpose, six foods (fish, vegetables, and cheese) were immersed in olive oil for 30 days and analyzed to determine these antioxidant phenols by LC–MS/MS. Oleuropein aglycone, hydroxytyrosol and tyrosol were the main phenols quantitatively enriched in the foods (up to 42.1, 26.2 and 53.0 mg/kg, respectively). The total phenolic content ranged from 5.8 to 12.1 mg in the evaluated foods taking as reference the recommended daily intake (150 g for fish, 200 g for vegetables, and 50 g for cheese). This research proves the phenolic enrichment of foods curated in olive oil, which can hypothetically increase their antioxidant and bioactive properties.

## Highlights

• Phenolic compounds were quantitatively transferred to foods curated in virgin olive oil.
• The main enriched phenols were hydroxytyrosol, tyrosol and oleuropein aglycone.
• Phenolic enrichment depends on the type of food and phenol.
• Intake of foods curated in EVOO provides a source of bioactive phenols.

## 1 Introduction

Traditionally, vegetable oils have been used to preserve food, mainly cheese, vegetables, meat, fish. This is a current preservation practice for these types of foods, which are commercially presented in vegetable oils, mainly, sunflower and olive oils. Pinto de Rezende, Barbosa, and Teixeira (2022) reviewed that some types of essential oils, with aromatic herbs and spices, could improve seafood preservation (Pinto de Rezende et al., 2022). In the Mediterranean area, the preferred vegetable oil used for food preservation is olive oil, which is particularly suitable for this purpose due to its antioxidant and antimicrobial properties (Shultz, Xu, & Buxbaum, 2008). These properties have been strongly linked to the presence of phenolic compounds (Cicerale, Lucas, & Keast, 2012).

Virgin Olive Oil (VOO) contains phenolic compounds. In the phenolic fraction of VOO, it is worth mentioning the presence of secoiridoid derivatives because of their high bioactivity (Servili et al., 2014). In fact, a health claim for the phenolic compounds of VOO has been recognized by the Commission Regulation (EU) 432/2012. This claim “olive oil phenols protect blood lipids against oxidative stress” could be used for those olive oils providing at least 5 mg of hydroxytyrosol, tyrosol and derivatives in a daily consumption of 20 g of olive oil (432/2012, 2012). Previous studies have revealed that VOOs have a high probability of containing at least 250 mg/kg of phenolic compounds responsible for the health claim (Criado-Navarro, López-Bascón, & Priego-Capote, 2020). Rubió et al. (2012) analyzed the effect of the intake of three different VOOs enriched with variable concentrations of the main phenolic families (secoiridoid derivatives, phenylalcohols and flavonoids). They found that a high intake of certain VOOs produced a dose-dependent response in the human systemic circulation due to phenolic content (Rubió et al., 2012). Additionally, Castillo-Luna, Ledesma-Escobar, Gómez-Díaz, and Priego-Capote (2022) confirmed that the variability of the phenolic urinary profile is related to the phenolic composition of consumed VOOs.

Food enrichment with bioactive components has been studied for a long time to improve nutraceutical properties for infants, adolescents, and women of childbearing age population who have special nutritional demands (Richardson, 1990). New technological advances have allowed the development of functional foods. These foods contain specific nutrients that provide health benefits (Kaur & Das, 2011). Kolanowski and Laufenberg (2006) demonstrated that enrichment of foods with polyunsaturated fatty acids (PUFAS) isolated from fish oil could increase the amount of PUFAs in our diet, being a benefit for our health (Kolanowski & Laufenberg, 2006). On the other hand, Cedola, Cardinali, Del Nobile, and Conte (2019) characterized a bread fortified with a high concentration of phenolic compound from olive paste (Cedola et al., 2019). In the same vein, tagliatelle pasta was enriched with phenols using grape pomace and olive pomace, obtaining 6.21 and 9 mg of phenols, respectively, in 100 g of pasta. Moreover, tagliatelle pasta maintained the phenolic compounds after cooking (Balli, Cecchi, Innocenti, Bellumori, & Mulinacci, 2021). Świeca, Sȩczyk, Gawlik-Dziki, and Dziki (2014) analyzed the positive increase of antioxidant capacity in bread enriched with quinoa leaves (Świeca et al., 2014). Furthermore, the addition of phenolic compounds to various foods such as potatoes, bread, meat or cheese improved the antioxidant activity, reduced the formation of acrylamide and N-ε-(carboxymethyl) lysine, and inhibited the oxidation of proteins, among other mechanisms (Kotsiou, Tasioula-Margari, Kukurová, & Ciesarová, 2010).

Artajo, Romero, Morelló, and Motilva (2006) studied the antioxidant activity of foods enriched with phenols according to their chemical structure. They observed beneficial effects when the enrichment was made with 3,4-dihydroxy (hydroxytyrosol, 3,4-dihydroxybenzoic acid, oleuropein, caffeic acid) and 3,4,5-trihydroxy (gallic acid) structures in an aromatic ring. In addition, they found that tyrosol had a lower antioxidant capacity due to the presence of a unique hydroxyl substituent (Artajo et al., 2006).

Notwithstanding these studies, the phenolic enrichment of foods curated in EVOO has not been investigated. The hypothesis here is that olive oil phenols could provide an added health value to curated food and increase its antioxidant capacity. Thus, the relevance of this research is that a widely accepted strategy for food preservation, which is even commercially implemented, can lead to phenolic enrichment and to improve the nutritional quality. In this research, we plan an experimental study involving the curation of six different foods in a high phenolic EVOO. The aims were: (i) to demonstrate the phenolic transfer from EVOO to six different foods when they are curated for 30 days; (ii) to identify the preferred phenols that enriched the foods; (iii) to study the kinetics of phenolic enrichment of foods; (iv) to quantitatively determine the transfer of EVOO phenolic compounds to foods.

## 2 Materials and methods

### 2.1 Chemicals and reagents

LC grade reagents and solvents were used in this research. Ammonium fluoride from Fisher Scientific (Hampton, NH, USA) was used as the ionizing agent. Methanol (MeOH) from Scharlab (Barcelona, Spain) and deionized water (18 mΩ • cm) from a Millipore Milli-Q water purification system (Merck Millipore, Bedford, MA, USA) were used for the preparation of chromatographic mobile phases.

Hydroxytyrosol and tyrosol were purchased from Extrasynthese (Genay, France), whereas secoiridoid derivatives oleacein and oleocanthal were acquired from Phytolab (Vestenbergsgreuth, Germany). On the other hand, oleuropein aglycone and ligstroside aglycone (both as monoaldehyde closed isomers) were purchased from TRC (Ontario, Canada). Syringaldehyde from Sigma-Aldrich (St. Louis, MO, USA) was used as an internal standard (IS) to control the LC-MS/MS performance.

### 2.2 Apparatus and instruments

An oscillating Vibromatic shaker and a Mixtasel-BL centrifuge from J.P. Selecta S.A.® (Barcelona, Spain), an Orto Alresa Digtor 21 centrifuge from Orto Alresa (Madrid, Spain) and a vortex shaker from IKA® (Wilmington, NC, USA) were used for sample preparation steps.

An Agilent 1200 series LC system (Palo Alto, CA, USA) with electrospray ionization (ESI) coupled to an Agilent 6410 triple quadrupole (QqQ) tandem mass spectrometer was used for the analysis of the prepared samples. For qualitative and quantitative analyses, MassHunter Workstation software (V—B.10, Agilent Technologies, Palo Alto, CA, USA) was used for data acquisition and processing.

### 2.3 Sample preparation of foods

200 g of cured cheese, soft cheese, salmon, cod, Cherry tomatoes and eggplants were purchased in a conventional supermarket in Cordoba (Spain). For this study, we selected an extra virgin olive oil (EVOO) with a similar concentration of aglycones (ligstroside aglycone and oleuropein aglycone), and oleacein and oleocanthal.

Each food was cut into ten cubes (1 cm^3^ pieces), placed in an amber glass container of 500 mL and covered with 250 mL of EVOO in the absence of light. In the case of fish (salmon and cod), the pieces were previously marinated by covering them with a mixture of sugar (50%) and salt (50%) for 48 h at refrigeration temperature (4 °C). Curation was monitored for 30 days by taking aliquots at days: 0 (blank, before immersion in EVOO), 1, 3, 5, 8, 11, 16, 21, 25 and 30. Aliquots were taken by taking 5 g of each food except for tomatoes, for which we took one unit weighing between 6 and 8 g. Food cubes were dried with a filter paper to completely remove residual EVOO. All food aliquots were directly frozen at −80 °C until they were processed for analysis.

Sample preparation was initiated by grinding with a mortar for homogenization. Then, 10 mL of 80:20 (*v*/v) MeOH:H_2_O with the IS (0.5 mg/L) was added and shaken for 10 min. The suspension was separated by centrifugation at 2900 ×*g* for 15 min, and the liquid phase was centrifuged again at 900 ×*g* for 8 min to remove suspended solids. Five consecutive extractions were performed on each food sample and two replicates were analyzed per sample.

### 2.4 Sample preparation of EVOO

Phenols were isolated by liquid-liquid extraction according to the protocol published protocol by Miho et al. (2018). Briefly, 250 μL of *n*-hexane was added to 0.5 g of oil and vortexed for 30 s. Subsequently, 2 mL of 80:20 MeOH:H_2_O with the IS (1 mg/L) was added and agitated for 2 min. Finally, the hydroalcoholic phase was isolated by centrifugation at 900 ×*g* for 8 min (Miho et al., 2018).

### 2.5 LC–MS/MS analysis

The chromatographic separation of phenolic compounds was carried out with a Mediterranea C_18_ (3 μm particle size, 5.0 × 0.46 cm i.d.) analytical column from Teknokroma (Barcelona, Spain). The column was protected with a Mediterranea C_18_ precolumn (4 μm particle size x 0. 30 cm i.d.). The precolumn and the column were maintained at 30 °C during analysis. The mobile phases were water (phase A) and methanol (phase B), both with 0.1% (*v*/v) formic acid as ionizing agent. A multiple reaction monitoring (MRM) method previously optimized by Miho et al. (2018) was used. The MRM parameters for the determination of the target phenols are listed in **Supplementary Table S1**. The ESI unit operated in negative ionization mode with temperature of 300 °C, nebulizer pressure of 50 psi, drying gas (N_2_) flow rate of 10 L/min, and capillary voltage of 3000 V (Miho et al., 2018). The injection volume for oil and food extract samples was 5 μL.

### 2.6 Quantitative determination of phenolic compounds

Calibration models were prepared by using refined sunflower oil spiked with multistandard phenolic solutions at variable concentrations (from 1 to 20 mg/kg). Spiked aliquots (1 g) were treated by liquid–liquid extraction in 2 mL 80:20 (v/v) MeOH:H_2_O solution. The extracts were analyzed by LC–MS/MS to obtain the calibration models (**Supplementary Table S2**) by using the ratio between the peak area of each phenol and that of corresponding IS.

### 2.7 Statistical analysis

*R studio* free software (version 4.2.3., http://www.r-project.org/) was used for processing and statistical analysis. Data normalization was performed by applying logarithms. The statistical analysis included a non-linear regression model and the analysis of successive differences with the MASS package based on a coding for factors (version 7.3–60., http://www.stats.ox.ac.uk/pub/MASS4/) to determine the optimal period as a function of the maximum concentration of each phenolic compound.

## 3 Results and discussion

### 3.1 Characterization of phenolic compounds in EVOO

In this research we selected an EVOO produced in Spain during the 2021 crop season. This EVOO was analyzed according to the method described by Miho et al. (2021). The selected EVOO contained a high concentration of secoiridoid derivatives. Thus, the sum of oleuropein and ligstroside aglycones was 344 mg/kg, while the addition of oleacein and oleocanthal was 482 mg/kg. With these concentration levels, we characterized the phenolic profile of this EVOO by following the parameters reported by Miho et al. (2021) in terms of the f and h factors. The f factor was 0.71, which means that oleacein and oleocanthal are slightly superior to the aglycone forms. The h factor was 1.43, indicating a predominance of hydroxytyrosol conjugated secoiridoids versus tyrosol conjugated secoiridoids (Table 1). This characterization is justified since secoiridoid derivatives are the dominant compounds in the phenolic profile of EVOO and allow the differentiation of cultivars according to their relative concentration (Miho et al., 2021). Complementary, simple phenols hydroxytyrosol and tyrosol were detected at concentrations below 10 mg/kg.

**Table 1:** Phenolic content of the EVOO selected for the study.

**Phenol**	**Concentration**⁎**(mg/kg)**
Oleocanthal	184
Oleacein	298
Oleuropein aglycone	191
Ligstroside aglycone	153
Hydroxytyrosol	7.1
Tyrosol	8.3
*f* factor	0.71
*h* factor	1.4

⁎ Variability in concentrations was always below 10% expressed as relative standard deviation.

### 3.2 Phenols detected in foods curated in EVOO

Foods selected for this research were dairy products (cured cheese and soft cheese), fish (salmon and cod) and vegetables (Cherry tomato and eggplant). Curation was monitored for 30 days which is considered the optimal processing time for this culinary technique. Aliquots were taken at 1, 3, 5, 8, 11, 16, 21, 25 and 30 days. Preliminary, the six foods were analyzed to confirm that none of them contained a detectable concentration of the target phenols prior to enrichment (day 0, blank samples). Therefore, the concentrations of phenols detected in the foods must be explained by enrichment due to curation in EVOO.

Qualitative evaluation of all foods analyzed at different days allowed the detection of hydroxytyrosol, tyrosol, oleuropein aglycone and ligstroside aglycone (**Supplementary Fig. S1**). Moreover, oleocanthal and oleacein were detected only in tomato. However, quantitative analysis was only feasible for the two simple phenols, hydroxytyrosol and tyrosol, and oleuropein aglycone. On the other hand, ligstroside aglycone, oleacein and oleocanthal were not found in significant concentrations for quantitation.

The qualitative comparison of the phenolic profiles showed that hydroxytyrosol was the major phenol in cured and soft cheese and salmon, followed by oleuropein aglycone and, finally, tyrosol. In addition, the two simple phenols dominated the phenolic enrichment in curated cod followed by oleuropein aglycone. Regarding vegetables (tomato and eggplant), oleuropein aglycone was the most enriched phenol followed by hydroxytyrosol and tyrosol. This could be explained by the presence of other phenolic families in vegetable foods that would contribute to preserve secoiridoids from hydrolysis and other transformations that occur in fish or cheese (Peschel et al., 2006; Vinson, Su, Zubik, & Bose, 2001). Hence, the presence of other phenols with antioxidant capacity would minimize the transformations of secoiridoids (Servili et al., 2014). **Supplementary Fig. S2** shows the mechanistic transformations occurring in oil during curation. The phenolic profile of EVOO is enriched in secoiridoids that are hydrolyzed during curation to release the simple phenolic structures hydroxytyrosol and tyrosol. These transformations seem to be more favored for oleacein and oleocanthal since these two phenols are detected in EVOO used for food curation at trace levels.

Despite residual olive oil was removed from food aliquots sampled during curation, it is mandatory to prove that the detection of phenols in food was explained by enrichment. For this purpose, we compared the phenolic profile of EVOO before curation with those collected after this process was completed for 30 days (Table 1, Table 2, respectively). This comparison revealed that olive oils after being used for curation were characterized by a low content of phenols. This reduced concentration was justified by a double effect: (1) the enrichment of foods, which is favored by its water content due to the hydrophilic nature of phenols, and (2) their involvement in oxidation reactions due to their antioxidant capacity, which contributes to the preservation of the food. Castillo-Luna et al. (2022) determined the degradation of phenolic compounds in EVOOs obtained from different cultivars stored for 12 months (Castillo-Luna, Criado-Navarro, Ledesma-Escobar, López-Bascón, & Priego-Capote, 2021). This degradation was less significant than that measured in olive oil after curation for 30 days. In addition, we compared the phenolic profiles detected in foods with those determined in the EVOO before curation. As previously mentioned, the main phenols detected in foods, even after curation for 24 h, were simple phenols and oleuropein aglycone. Hydroxytyrosol and tyrosol were the less abundant phenols in EVOO (<10 mg/kg). Therefore, hydrolysis reactions of secoiridoids and the enrichment of foods may explain the predominance of simple phenols in curated foods as hydroxytyrosol and tyrosol are the most hydrophilic compounds of the EVOO phenolic profile.

**Table 2:** Phenolic content determined in the EVOO after curation of different foods.

**Phenol**	**Concentration**⁎**, mg/kg**					
	**Salmon**	**Cod**	**Soft cheese**	**Cured cheese**	**Eggplant**	**Tomato**
Oleocanthal	0.04	0.97	1.5	1.7	0	17.7
Oleacein	0.72	0.73	2.5	3.9	0.61	244
Oleuropein aglycone	6.0	16.1	4.1	4.8	2.3	8.3
Ligstroside aglycone	5.5	15.6	3.6	4.3	1.8	9.2
Hydroxytyrosol	2.0	1.1	5.0	1.2	1.8	4.0
Tyrosol	7.4	11	4.6	1.2	7.2	4.4

⁎ Variability in concentrations was always lower than 10% expressed as relative standard deviation.

### 3.3 Enrichment factor and kinetics

The transfer of phenolic compounds from EVOO to curated foods was monitored over a period of 30 days by analyzing ten aliquots sampled on different days. The goal was to find out when the maximum enrichment level was reached in each food. Five extractions were carried out in each aliquot to ensure a quantitative extraction. Analysis of all aliquots indicated that three extraction steps were required to achieve efficiencies of around 80% or higher in the six evaluated foods (Table 3). The efficiency for tyrosol isolation ranged from 87.1 to 99.9%, for oleuropein aglycone from 78.6 to 93.9%, and for hydroxytyrosol from 79.5 to 91.4%. These results are crucial to ensure the quantitative determination of phenolic compounds in enriched foods.

**Table 3:** Extraction efficiency determined with three consecutive steps. The reference was determined by considering five extraction steps.

**Average factor in percentage**	**Oleuropein aglycone**	**Hydroxytyrosol**	**Tyrosol**
Salmon	80.1	79.9	91.7
Cod	78.6	79.5	96.8
Eggplant	93.9	82.1	97.5
Tomato	81.4	91.4	99.9
Cured cheese	81.2	81.8	87.1
Soft cheese	80.1	82.0	92.0

**Fig. 1:**
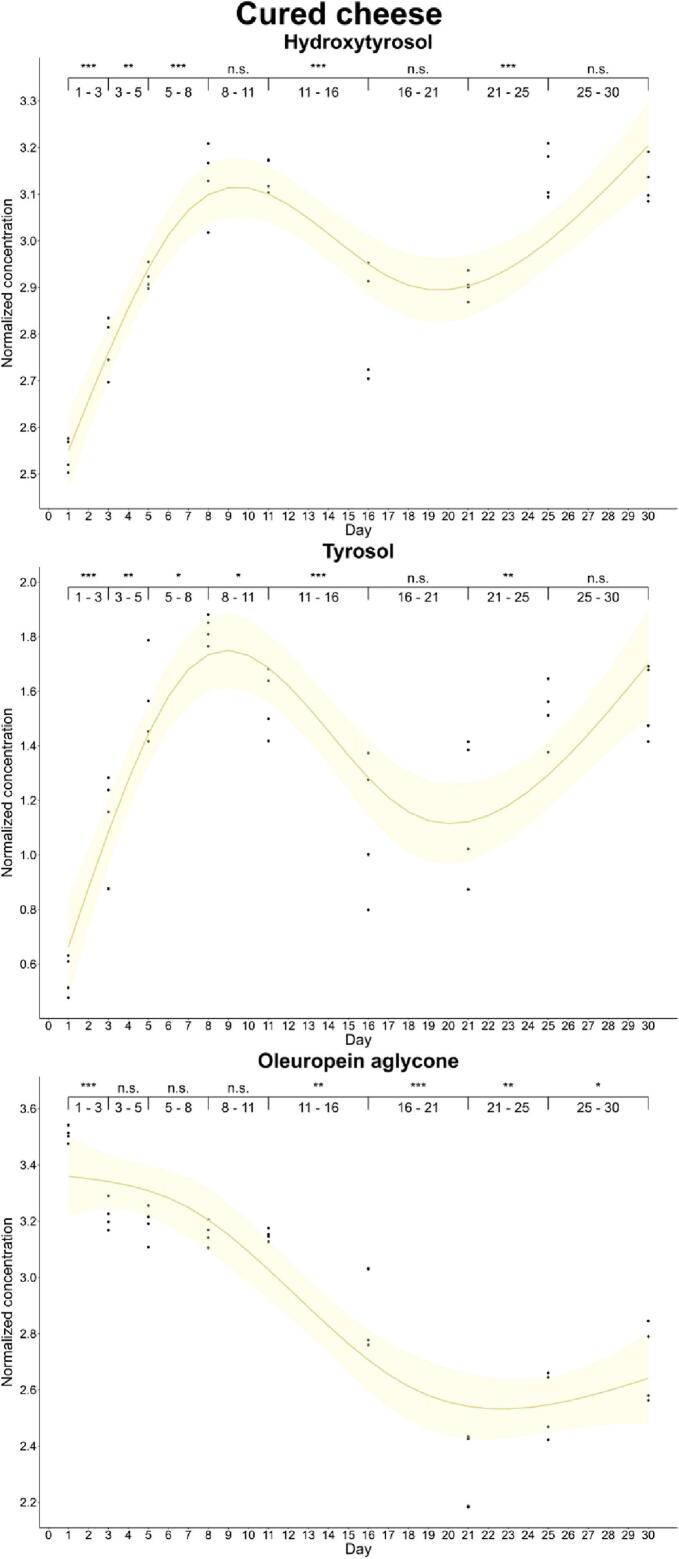
Kinetics of the phenolic enrichment of hydroxytyrosol, tyrosol and oleuropein aglycone of cured cheese in EVOO. Significant differences were determined by the successive difference contrast test and are labeled as “***p-value < 0.001”, “**p-value: 0.001–0.01”, “*p-value: 0.01–0.05” and “n.s. *p*-value > 0.05”. Concentrations were normalized before statistical analysis.

**Fig. 2:**
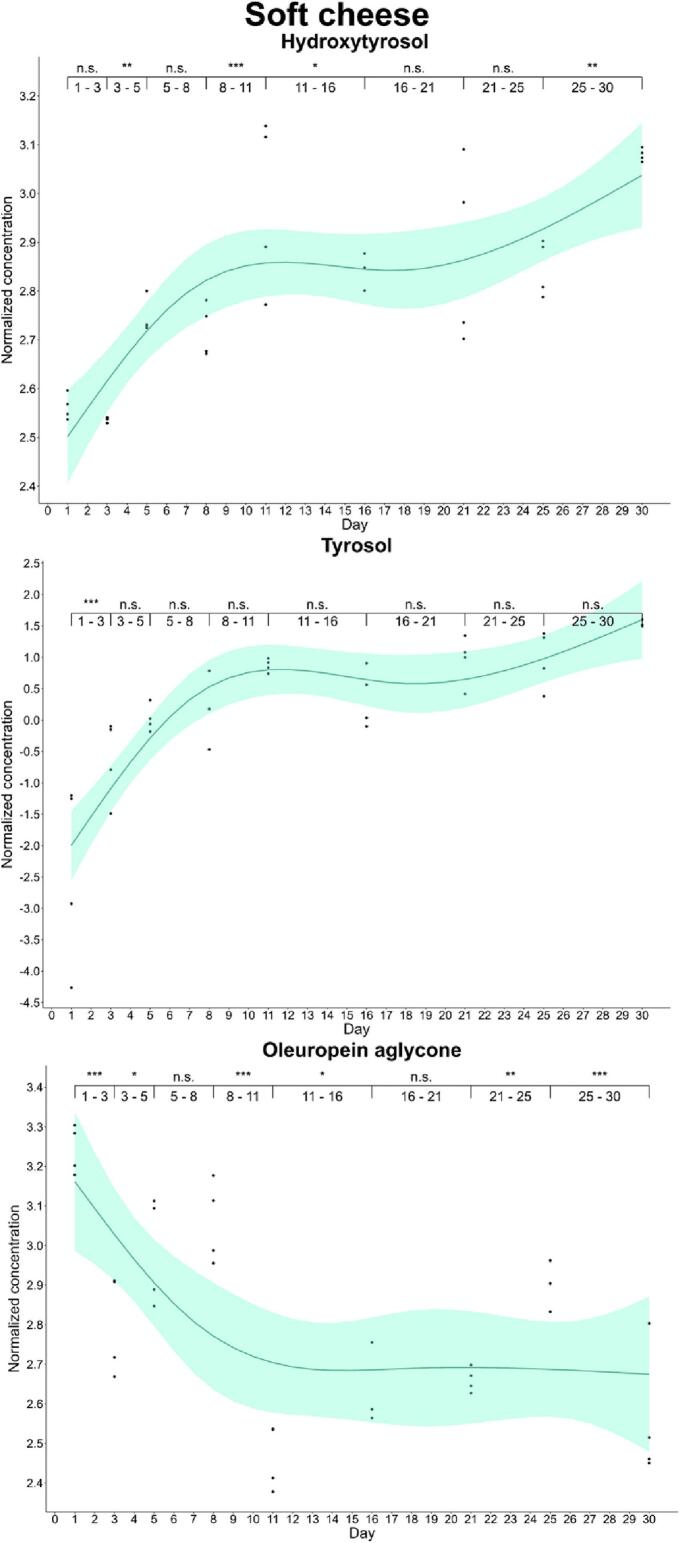
Kinetics of the phenolic enrichment of hydroxytyrosol, tyrosol and oleuropein aglycone of soft cheese in EVOO. Significant differences were determined by the successive difference contrast test and are labeled as “***p-value < 0.001”, “**p-value: 0.001–0.01”, “*p-value: 0.01–0.05” and “n.s. *p*-value > 0.05”. Concentrations were normalized before statistical analysis.

Furthermore, we completed enrichment kinetics for each food during the monitored period. We observed differences in the enrichment kinetics of phenols and foods by analysis of successive differences. Thus, oleuropein aglycone reached the maximum enrichment level (*p*-value<0.001) in cheese on day 1 (33.4 and 25.6 mg/kg in cured and soft cheese, respectively) and then, its concentration showed a significant decreasing trend. On the other hand, the hydroxytyrosol content increased until day 8 in cured cheese (22.9 mg/kg), while this simple phenol showed a gradual increase in soft cheese during the curation process, reaching the highest concentration (*p*-value: 0.001–0.01) on day 30 (21.8 mg/kg). This increase is explained by the decrease in the levels of oleuropein aglycone, which could be acting as antioxidant releasing the simple phenolic structure by hydrolysis. A particular case was that of tyrosol, since its concentration in soft cheese increased gradually up to day 30, while we observed a significant decline at day 11 in cured cheese (Fig. 1, Fig. 2).

**Fig. 3:**
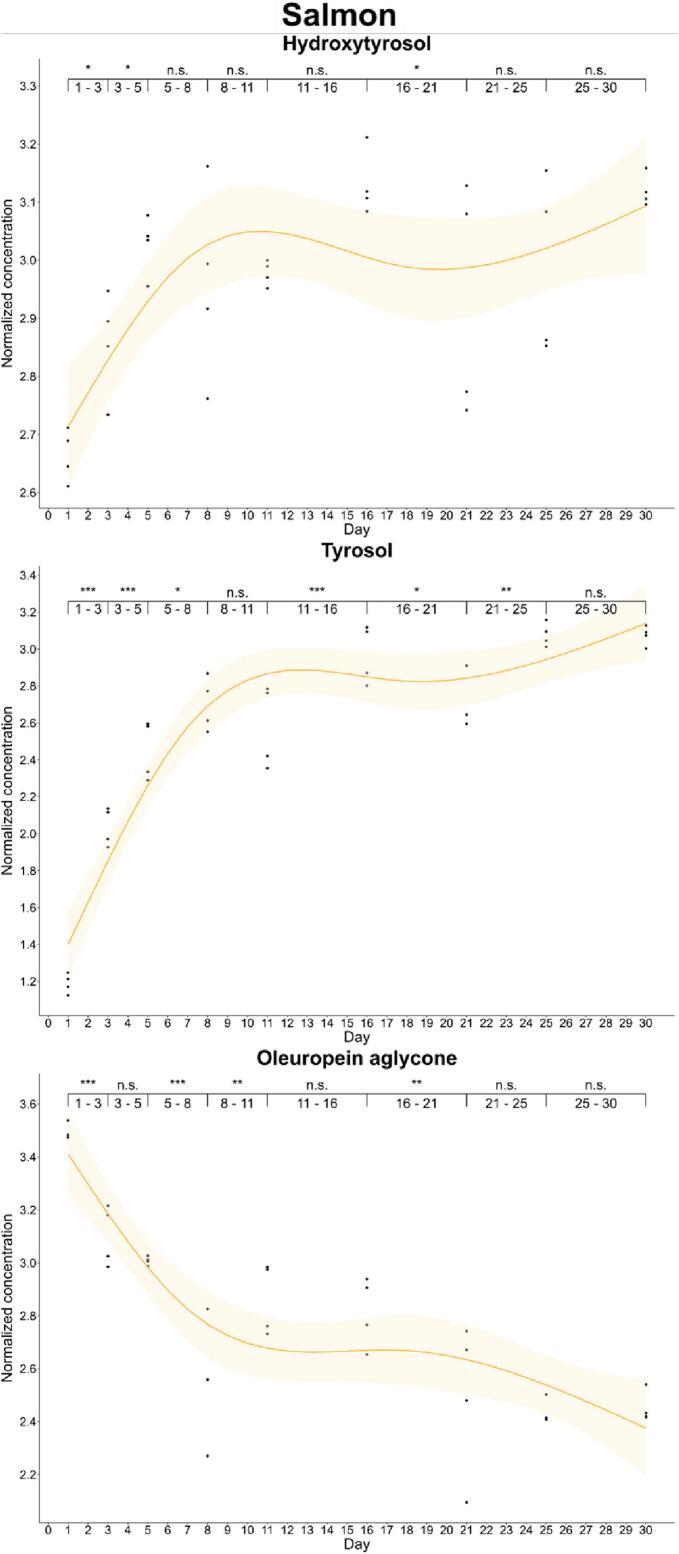
Kinetics of the phenolic enrichment of hydroxytyrosol, tyrosol and oleuropein aglycone of salmon in EVOO. Significant differences were determined by the successive difference contrast test and are labeled as “***p-value < 0.001”, “**p-value: 0.001–0.01”, “*p-value: 0.01–0.05” and “n.s. *p*-value > 0.05”. Concentrations were normalized before statistical analysis.

**Fig. 4:**
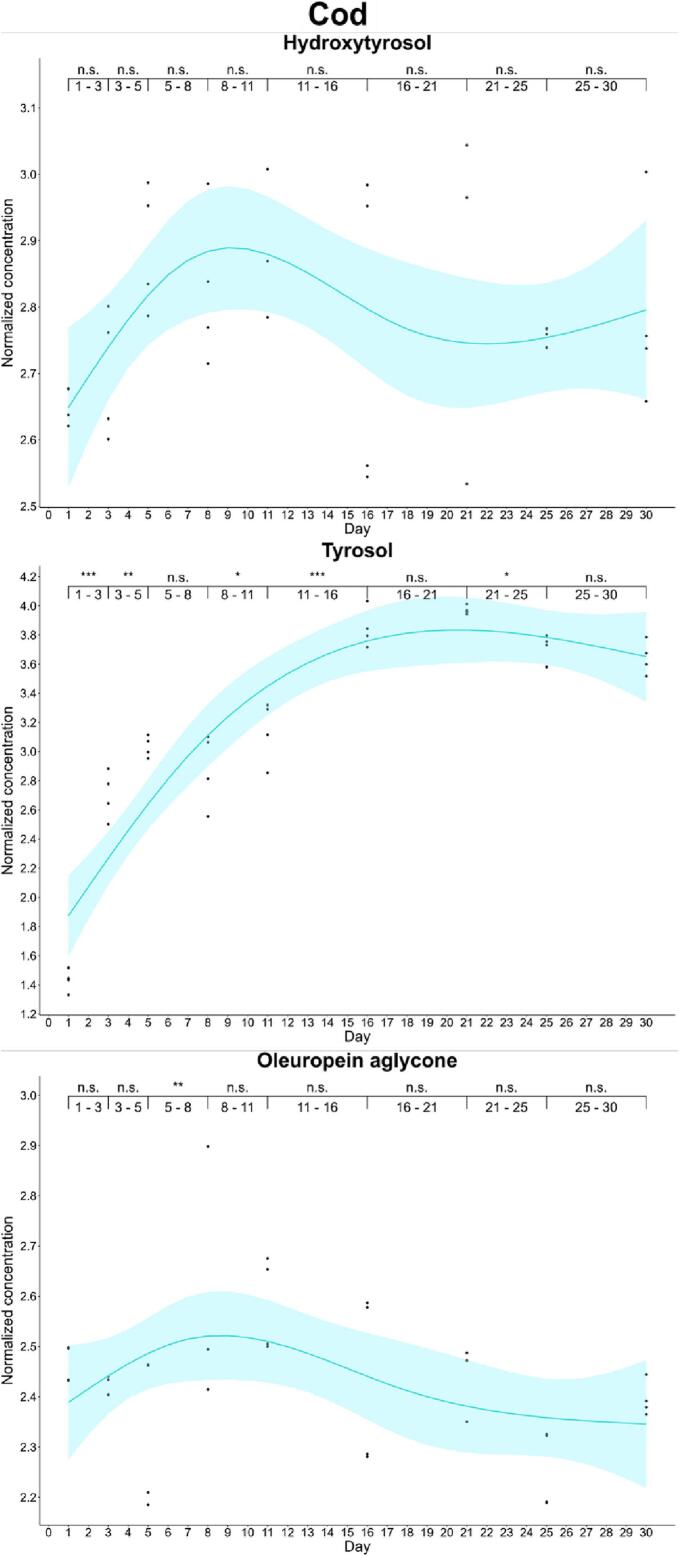
Kinetics of the phenolic enrichment of hydroxytyrosol, tyrosol and oleuropein aglycone of cod in EVOO. Significant differences were determined by the successive difference contrast test and are labeled as “***p-value < 0.001”, “**p-value: 0.001–0.01”, “*p-value: 0.01–0.05” and “n.s. *p*-value > 0.05”. Concentrations were normalized before statistical analysis.

We also found differences in the enrichment kinetics of fish. In salmon, oleuropein aglycone was enriched to a maximum on day 1 (33.0 mg/kg) and then, its concentration decreased up to stabilization. In cod, the maximum enrichment of oleuropein aglycone was also found on day 6 (13.4 mg/kg), but the concentration of this secoiridoid did not decrease significantly during the first three weeks. Hydroxytyrosol showed a different pattern in salmon and cod. Thus, we observed a non-significant increase in the concentration of hydroxytyrosol in cod. In contrast, hydroxytyrosol experienced a first increase at day 5 (*p*-value: 0.01–0.05) and at day 16 (16.1 mg/kg). Complementary, tyrosol reported a progressive accumulation in both cod and salmon during the whole curation study. This increase justifies that tyrosol conjugated secoiridoids such as oleocanthal and ligstroside aglycone were not detected in curated fish (Fig. 3, Fig. 4).

**Fig. 5:**
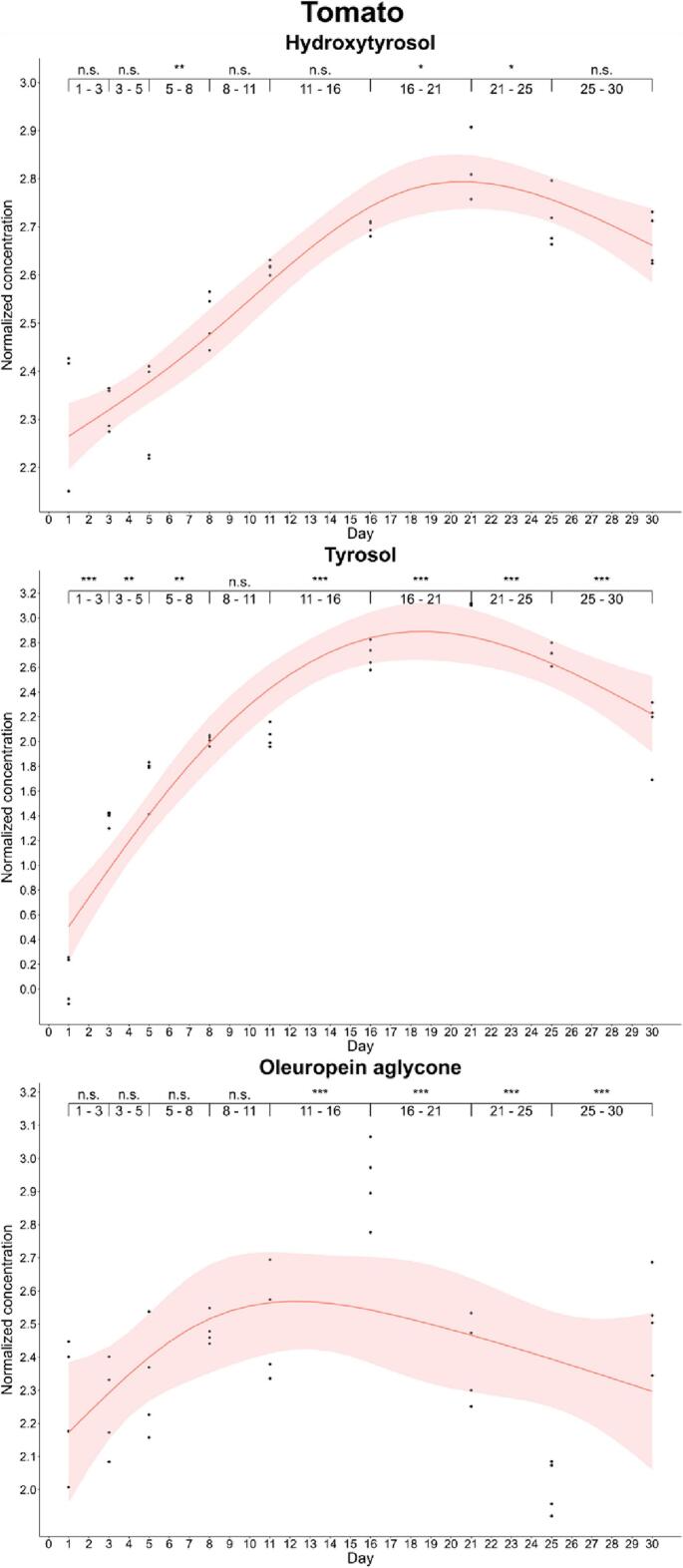
Kinetics of the phenolic enrichment of hydroxytyrosol, tyrosol and oleuropein aglycone of tomato in EVOO. Significant differences were determined by the successive difference contrast test and are labeled as “***p-value < 0.001”, “**p-value: 0.001–0.01”, “*p-value: 0.01–0.05” and “n.s. *p*-value > 0.05”. Concentrations were normalized before statistical analysis.

**Fig. 6:**
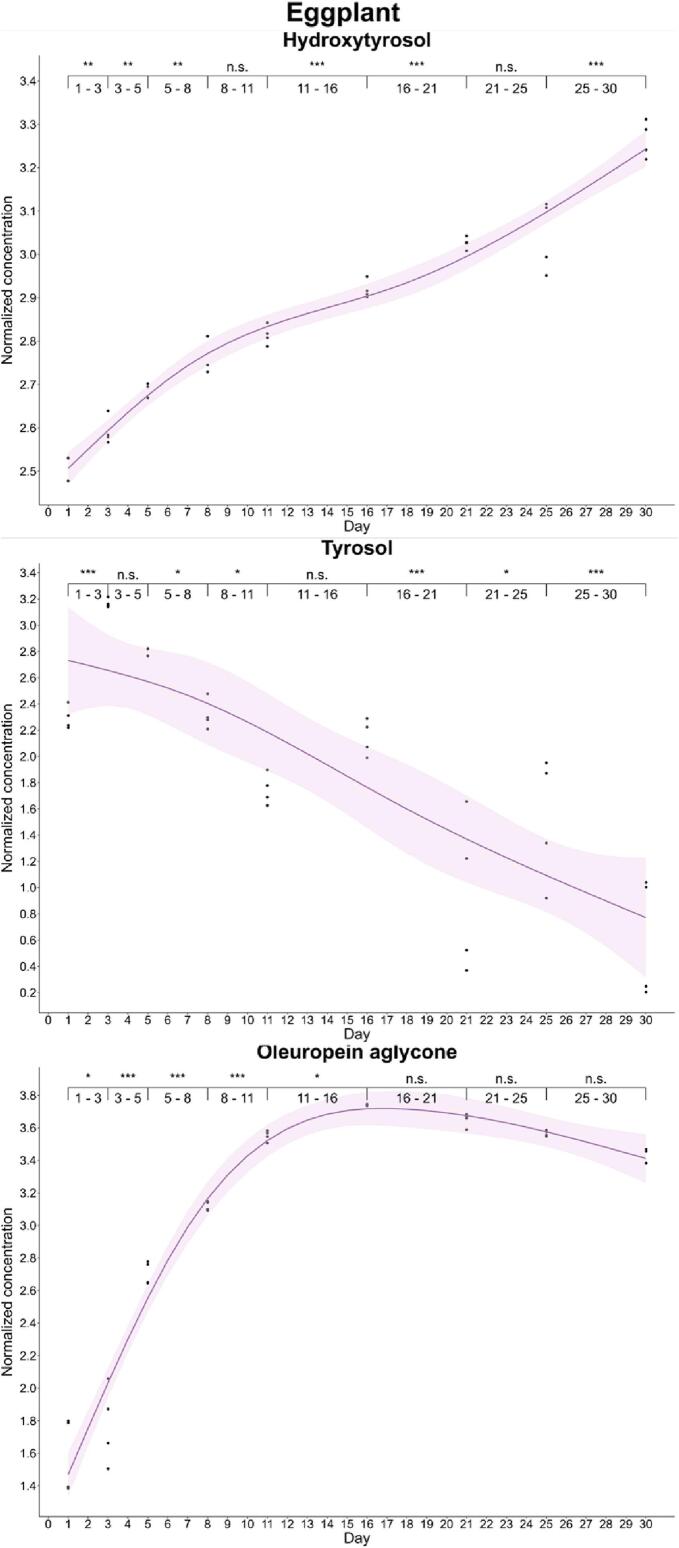
Kinetics of the phenolic enrichment of hydroxytyrosol, tyrosol and oleuropein aglycone of eggplant in EVOO. Significant differences were determined by the successive difference contrast test and are labeled as “***p-value < 0.001”, “**p-value: 0.001–0.01”, “*p-value: 0.01–0.05” and “n.s. *p*-value > 0.05”. Concentrations were normalized before statistical analysis.

Enrichment of phenols in vegetables was also variable. Thus, oleuropein aglycone and hydroxytyrosol were efficiently enriched in tomato and eggplant. The maximum enrichment level of oleuropein aglycone was found at day 16 in both vegetables (18.8 and 42.1 mg/kg in tomato and eggplant, respectively), while hydroxytyrosol reported a slight difference, since its maximum level was detected at days 21 and 30 in tomato and eggplant, respectively. Substantial differences were found in the case of tyrosol because the maximum transfer was detected on day 21 in tomato (22.4 mg/kg), while in eggplant this was found at day 3 (23.7 mg/kg). In both vegetables, a significant decrease was described after detection of the maximum transfer (Fig. 5, Fig. 6).

### 3.4 Phenolic enrichment of curated foods

The enrichment of foods with phenolic compounds constitutes a strategy to increase their health value due to the bioactive properties of phenols. Since the phenolic enrichment of foods by curation in EVOO has been demonstrated in this research, a quantitative determination of phenols in foods is mandatory, taking as reference the recommended daily intake of each food (Table 4) (Spanish Agency for Food Safety and Nutrition, 2022). Thus, the recommended daily intake of fish is 150 g. With this estimation, a daily consumption of salmon and cod curated in EVOO could provide 8.9 and 12.1 mg of phenolic compounds (hydroxytyrosol, tyrosol and oleuropein aglycone) per 150 g of intake. These maximum levels were reached on day 16 in salmon and cod, respectively (**Supplementary Fig. S3**).

**Table 4:** Healthy portions according to the food groups as defined by the Spanish Agency for Food Safety and Nutrition (AESAN).

**Food group**	**Selected food**	**Weight of each portion**	**Recommended frequency**
Vegetables	Eggplant	150–200 g	≥ 2 portion per day
	Tomato		
Fish	Salmon	125–150 g	3–4 portion per week
	Cod		
Dairy	Cured cheese	40–60 g	2–4 portion per day
	Soft cheese	80–125 g	

The recommended daily intake of vegetables is 200 mg (Table 4). The two tested vegetables reported a similar pattern to those observed in fish. Thus, the recommended daily intake of tomato and eggplant curated in EVOO provides a total phenolic content of 7.3 and 10.4 mg for tomato and eggplant, respectively. These levels were also reached on day 16 (*p*-value <0.001) in both eggplant and tomato. The comparison of the enrichment kinetics allowed to conclude that phenolic transfer followed a similar trend in both vegetables, but this was favored in eggplant (**Supplementary Fig. S4**).

Cured and soft cheeses described some differences by considering a recommended daily intake of 50 g. Soft cheese was optimally enriched significantly (*p*-value <0.001) at day 5, containing 5.8 mg of total phenolic compounds in 50 g. After the first day, the phenolic content was always below 6 mg/50 g daily intake, and no significant differences were found on the monitored days. Regarding cured cheese, the enrichment was higher as compared to soft cheese. Thus, a daily intake of 50 g of cured cheese contains 7.9 mg of hydroxytyrosol, tyrosol and oleuropein aglycone at day 8 (**Supplementary Fig. S5**).

According to the Commission Regulation 432/2012, a health claim could be attributed to olive oils providing at least 5 mg of hydroxytyrosol, tyrosol and derivatives in 20 g of product per daily consumption (432/2012, 2012). Under these conditions, the health claim declares that “Olive oil phenols contribute to the protection of blood lipids from oxidative stress”. The health claim, named “Polyphenols of olive oil” is specific to olive oil since the precursors of this claim are only found in this vegetable oil. With these premises, an equivalence can be established by considering the health claim related to phenolic compounds of olive oil. **Supplementary Figs. S3, S4 and S5** show how all foods curated in this research were enriched in phenolic compounds at concentrations above 5.0 mg/recommended daily intake. This enrichment even exceeded 10 mg in cod or eggplant. Despite no studies have been proposed about the bioavailability of phenolic compounds in foods curated in EVOO, their inclusion in the diet would represent a relevant contribution of phenols. These results take on an additional interest since these foods are included as main pillars in the Mediterranean diet.

## 4 Conclusions

This research confirms the phenolic enrichment of different types of foods curated in EVOO. Two simple phenols, tyrosol and hydroxytyrosol, and oleuropein aglycone were the main phenols enriched in foods, proving that the initial phenolic profile of EVOO is chemically modified during curation to avoid food decomposition.

Complementarily, oleuropein aglycone presented a similar behavior in fish and cheese since its concentration was higher during the first days of curation. By contrast, the concentration of oleuropein aglycone increased until day 16 in both studied vegetables. Furthermore, hydroxytyrosol showed superior concentrations from the second half of the curation period, being similar in fish, vegetables, and cheeses. On the other hand, tyrosol concentration followed the same behavior as hydroxytyrosol in the case of fish and cheese. Tyrosol concentration decreased from day 21 and 3 in tomato and eggplant, respectively.

This research proves the phenolic enrichment of food curated in EVOO. This preservation technique, which is commercially implemented, can be a strategy to improve the health benefits and antioxidant capacity of food.

## CRediT authorship contribution statement

**A. Castillo-Luna:** Writing – review & editing, Writing – original draft, Validation, Methodology, Investigation, Formal analysis, Conceptualization. **F. Priego-Capote:** Writing – review & editing, Writing – original draft, Validation, Methodology, Investigation, Funding acquisition, Formal analysis, Conceptualization.

## Declaration of competing interest

The authors declare that they have no known competing financial interests or personal relationships that could have appeared to influence the work reported in this paper.

## Data Availability

Data will be made available on request.

## Appendix A Supplementary data

**Supplementary Figure S1.** MRM chromatograms of phenolic compounds detected in the six foods curated in EVOO. Chromatograms for curated foods are shown in different colors while blank foods are shown in black. (1) hydroxytyrosol, (2) tyrosol, (3) oleuropein aglycone, (4) ligstroside aglycone, (5) oleacein and (6) oleocanthal. **Supplementary Figure S2.** Mechanistic pathway that shows the main transformations occurring to phenolic compounds of EVOO. Thick arrows represent the most favored transformations to simple phenols hydroxytyrosol and tyrosol. **Supplementary Figure S3.** Total phenolic content expressed as mg/g of daily intake of salmon and cod curated in EVOO. Significant differences were determined by the successive difference contrast test and are labeled as “****p*-value < 0.001”, “***p*-value: 0.001-0.01”, “**p*-value: 0.01-0.05” and “n.s. *p*-value > 0.05”. **Supplementary Figure S4.** Total phenolic content expressed as mg/g of daily intake of tomato and eggplant curated in EVOO. Significant differences were determined by the successive difference contrast test and are labeled as “****p*-value < 0.001”, “***p*-value: 0.001-0.01”, “**p*-value: 0.01-0.05” and “n.s. *p*-value > 0.05”. **Supplementary Figure S5.** Total phenolic content expressed as mg/g of daily intake of soft and cured cheese curated in EVOO. Significant differences were determined by the successive difference contrast test and are labeled as “****p*-value < 0.001”, “***p*-value: 0.001-0.01”, “**p*-value: 0.01-0.05” and “n.s. *p*-value > 0.05”. Image 1
