# Supplementary material for: Phenolic enrichment of foods curated in olive oil: Kinetics and chemical evaluation
Source: Food Chem X. 2024 Apr 22;22:101398. doi: 10.1016/j.fochx.2024.101398 (PMC11061228; doi:10.1016/j.fochx.2024.101398)

**Supplementary Table 1.** LC–MS/MS parameters for determination of phenolic compounds.

| Compound | RT*  (min) | Precursor ion  (m/z) | Product ion  (m/z) | Fragmentor | Collision energy  (eV) |
| --- | --- | --- | --- | --- | --- |
| Hydroxytyrosol | 2.0 | 153.1 | 123.1 | 40 | 15 |
| Tyrosol | 2.2 | 137.1 | 106.1 | 80 | 15 |
| Syringaldehyde (IS) | 2.7 | 181.1 | 151.1 | 40 | 15 |
| Oleacein | 3.4 | 319.2 | 69.3 | 80 | 20 |
| Oleocanthal | 5.3 | 303.1 | 59.3 | 80 | 15 |
| Oleuropein aglycone | 6.3 | 377.2 | 275.1 | 20 | 20 |
| Ligstroside aglycone | 6.4 | 361.1 | 291.1 | 60 | 15 |

**Retention time.*

**Supplementary Table 2.** Calibration models prepared for quantitative analysis of target phenols.

| **Compound** | **Calibration model** | **R2*** | **Calibration range** |
| --- | --- | --- | --- |
| Hydroxytyrosol | y = 1.9728x ± 0.0466 – 0.3116 ± 0.1199 | 0.9956 | 1 – 20 mg/kg |
| Tyrosol | y = 0.178x ± 0.0056 + 0.0004 ± 0.0142 | 0.9924 | 1 – 20 mg/kg |
| Oleacein | y = 0.9373x ± 0.0214 – 0.1332 ± 0.0552 | 0.9958 | 1 – 20 mg/kg |
| Oleocanthal | y = 1.0275x ± 0.0347 + 0.0174 ± 0.0893 | 0.9910 | 1 – 20 mg/kg |
| Oleuropein aglycone | y = 1.3191x ± 0.0511 + 0.0456 ± 0.1316 | 0.9881 | 1 – 20 mg/kg |
| Ligstroside aglycone | y = 5.0127x ± 0.2337 + 0.6737 ± 0.6016 | 0.9829 | 1 – 20 mg/kg |

**Regression coefficient.*

**Supplementary Figure legends**

**Supplementary Figure S1.** MRM chromatograms of phenolic compounds detected in the six foods curated in EVOO. Chromatograms for curated foods are shown in different colors while blank foods are shown in black. (1) hydroxytyrosol, (2) tyrosol, (3) oleuropein aglycone, (4) ligstroside aglycone, (5) oleacein and (6) oleocanthal.

**Supplementary Figure S2.** Mechanistic pathway that shows the main transformations occurring to phenolic compounds of EVOO. Thick arrows represent the most favored transformations to simple phenols hydroxytyrosol and tyrosol.

**Supplementary Figure S3.** Total phenolic content expressed as mg/g of daily intake of salmon and cod curated in EVOO. Significant differences were determined by the successive difference contrast test and are labeled as “****p*-value < 0.001”, “***p*-value: 0.001-0.01”, “**p*-value: 0.01-0.05*”*and “n.s. *p*-value > 0.05”.

**Supplementary Figure S4.** Total phenolic content expressed as mg/g of daily intake of tomato and eggplant curated in EVOO. Significant differences were determined by the successive difference contrast test and are labeled as “****p*-value < 0.001”, “***p*-value: 0.001-0.01”, “**p*-value: 0.01-0.05*”*and “n.s. *p*-value > 0.05”.

**Supplementary Figure S5.** Total phenolic content expressed as mg/g of daily intake of soft and cured cheese curated in EVOO. Significant differences were determined by the successive difference contrast test and are labeled as “****p*-value < 0.001”, “***p*-value: 0.001-0.01”, “**p*-value: 0.01-0.05*”*and “n.s. *p*-value > 0.05”.

**Supplementary Figure S1.**


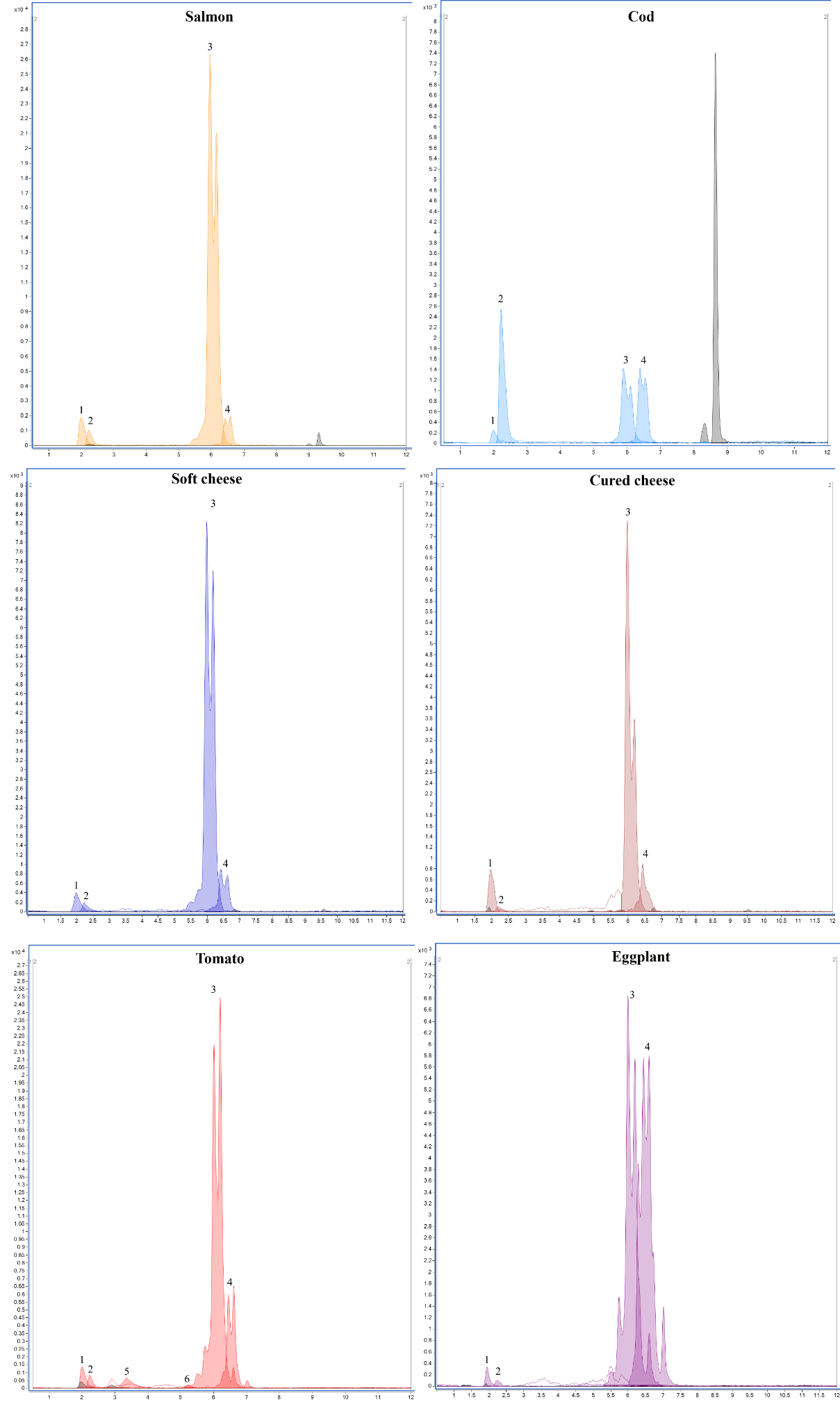


**Supplementary Figure S2.**

**
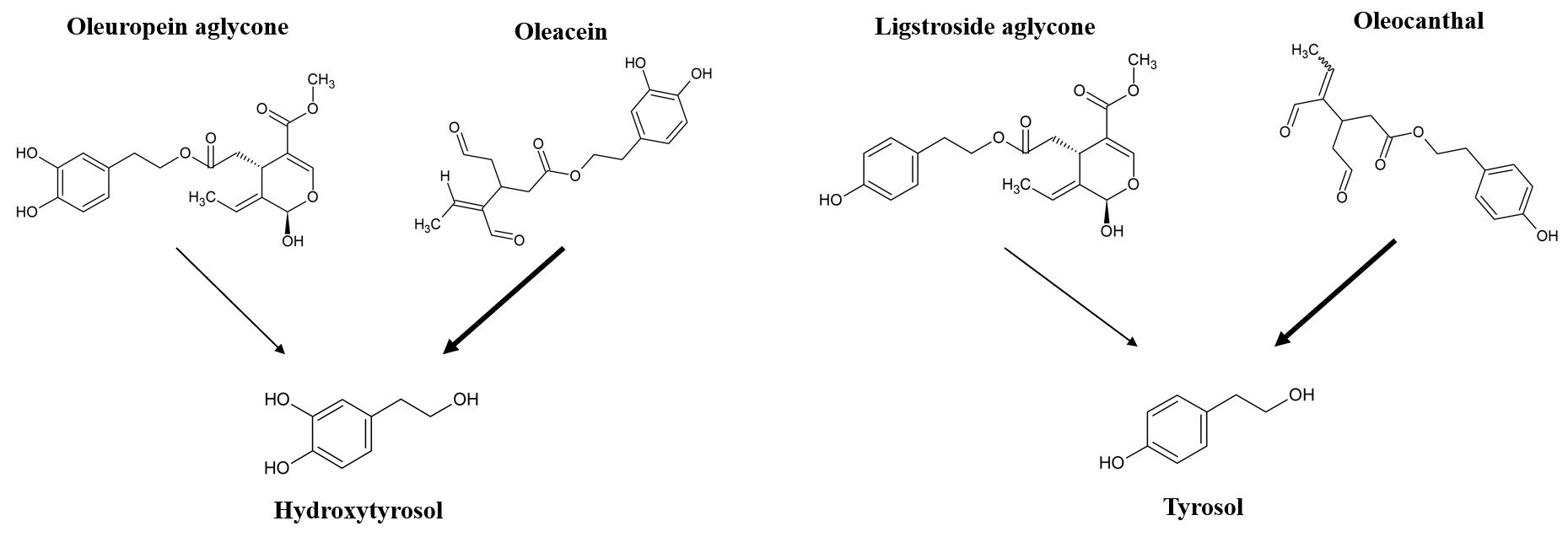
**

**Supplementary Figure S3.**


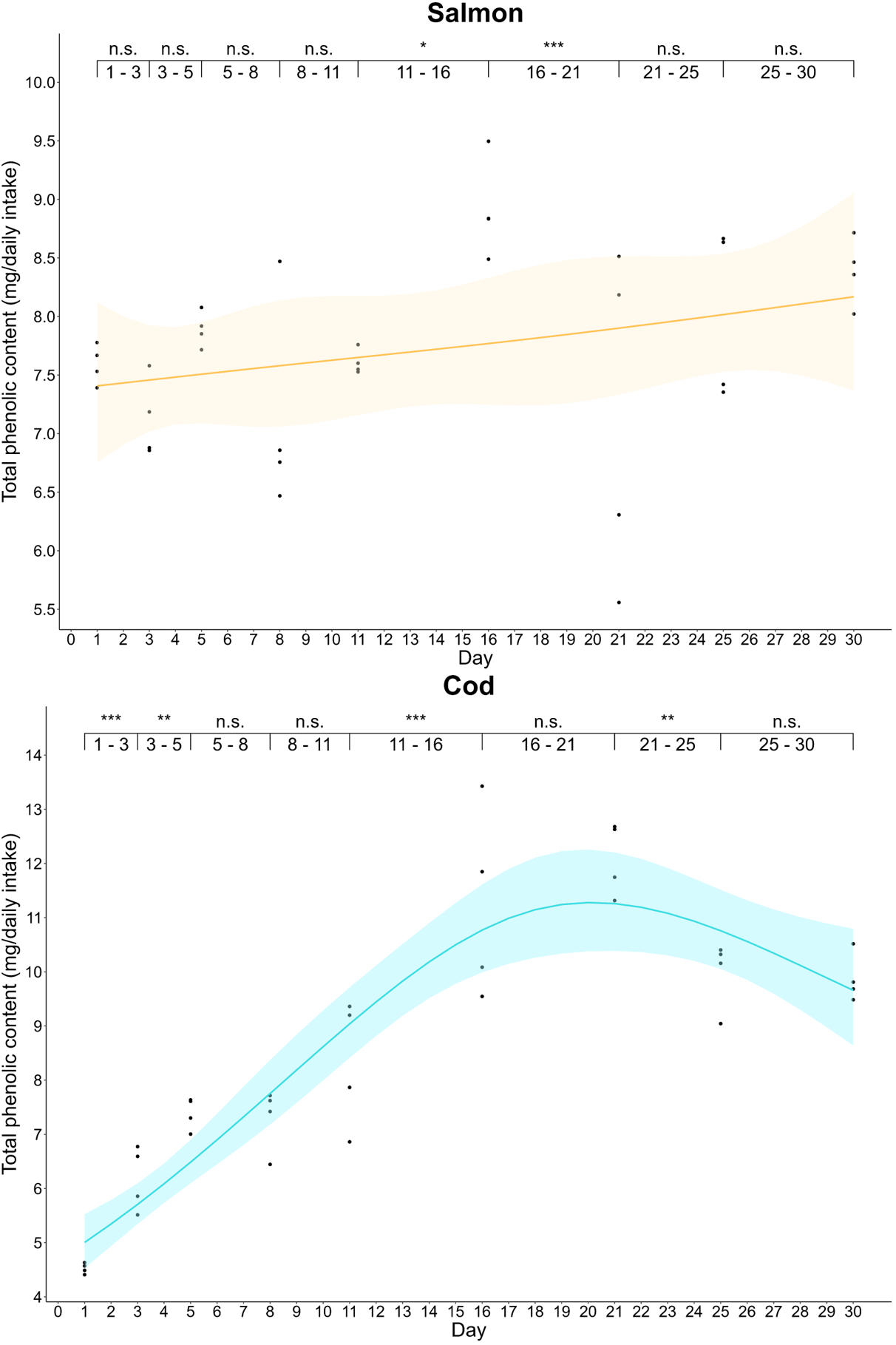


**Supplementary Figure S4.**


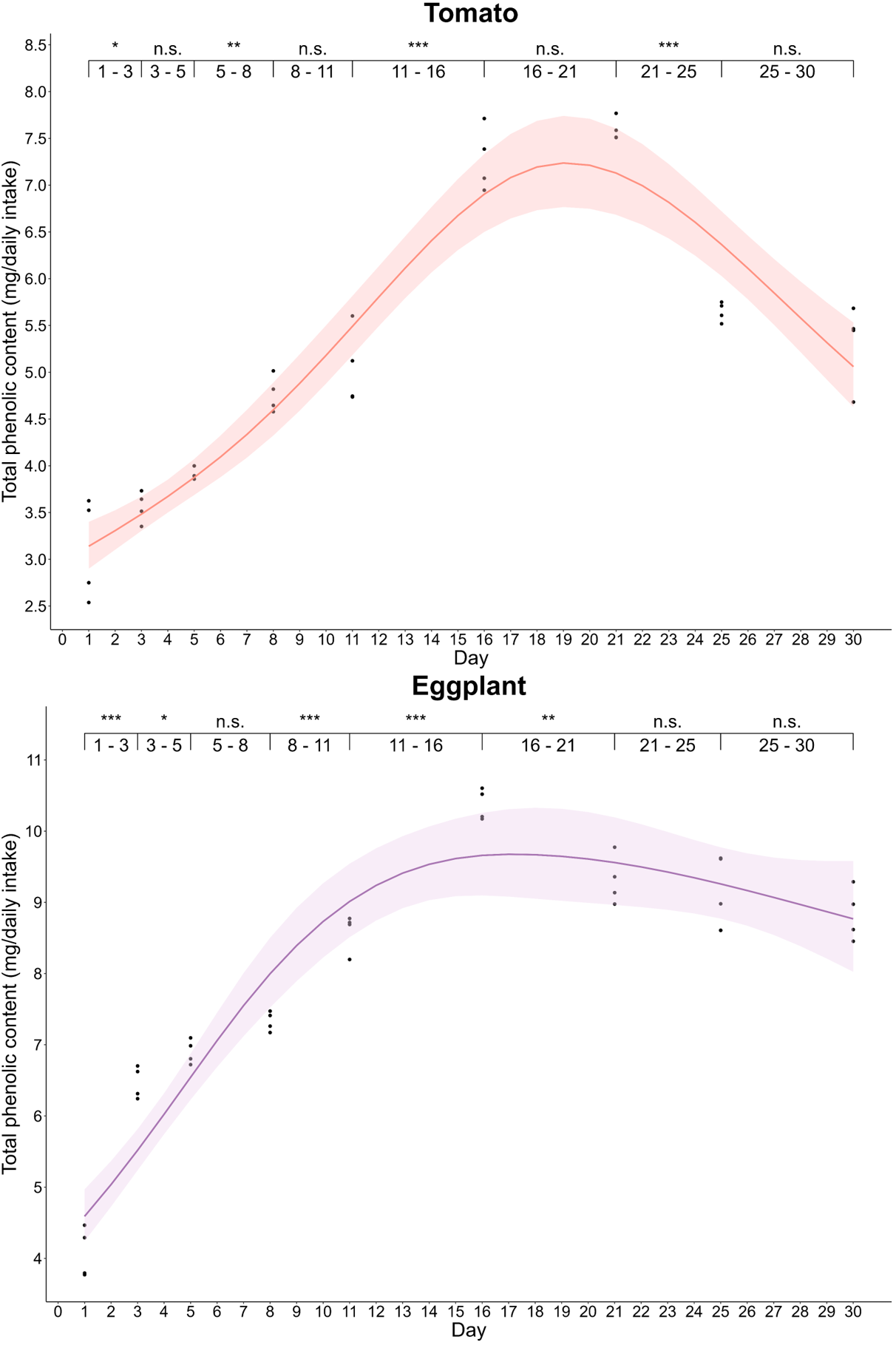


**Supplementary Figure S5.**


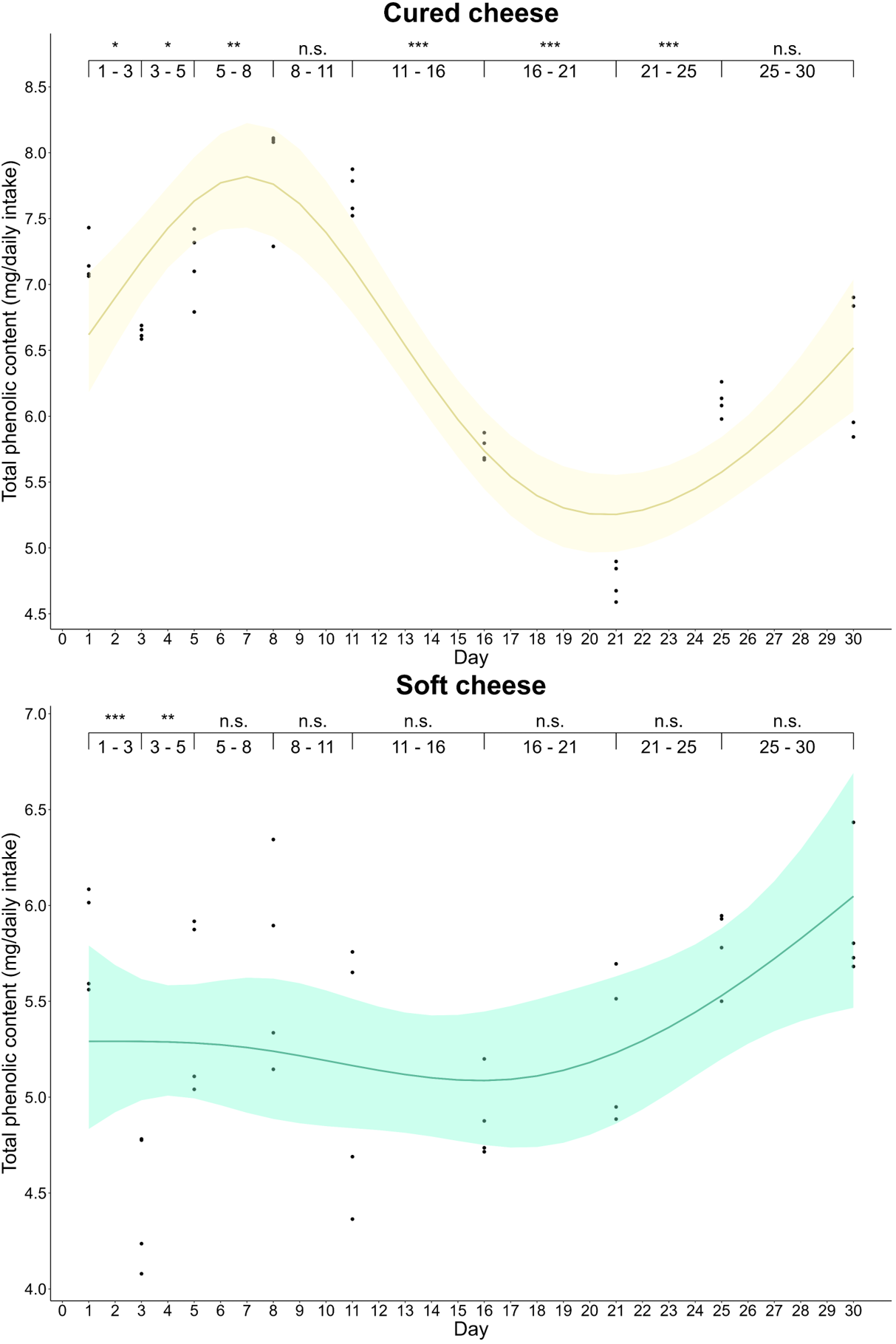

Supplement: Supplementary file 1 — Supplementary Figure S1. MRM chromatograms of phenolic compounds detected in the six foods curated in EVOO. Chromatograms for curated foods are shown in different colors while blank foods are shown in black. (1) hydroxytyrosol, (2) tyrosol, (3) oleuropein aglycone, (4) ligstroside aglycone, (5) oleacein and (6) oleocanthal. Supplementary Figure S2. Mechanistic pathway that shows the main transformations occurring to phenolic compounds of EVOO. Thick arrows represent the most favored transformations to simple phenols hydroxytyrosol and tyrosol. Supplementary Figure S3. Total phenolic content expressed as mg/g of daily intake of salmon and cod curated in EVOO. Significant differences were determined by the successive difference contrast test and are labeled as “***p-value < 0.001”, “**p-value: 0.001-0.01”, “*p-value: 0.01-0.05” and “n.s. p-value > 0.05”. Supplementary Figure S4. Total phenolic content expressed as mg/g of daily intake of tomato and eggplant curated in EVOO. Significant differences were determined by the successive difference contrast test and are labeled as “***p-value < 0.001”, “**p-value: 0.001-0.01”, “*p-value: 0.01-0.05” and “n.s. p-value > 0.05”. Supplementary Figure S5. Total phenolic content expressed as mg/g of daily intake of soft and cured cheese curated in EVOO. Significant differences were determined by the successive difference contrast test and are labeled as “***p-value < 0.001”, “**p-value: 0.001-0.01”, “*p-value: 0.01-0.05” and “n.s. p-value > 0.05”. [file mmc1.docx]
